# Integrating research in health professions education: a scoping review

**DOI:** 10.1186/s12909-023-04615-4

**Published:** 2023-09-08

**Authors:** Kirsti Riiser, Runa Kalleson, Heidi Holmen, Astrid Torbjørnsen

**Affiliations:** 1 Department of Rehabilitation Science and Health Technology, Faculty of Health Sciences, OsloMet - Oslo Metropolitan University, PO Box 4, St. Olavsplass, Oslo, N-0130 Norway; 2 Department of Nursing and Health Promotion, Faculty of Health Sciences, OsloMet - Oslo Metropolitan University, Oslo, Norway; 3 Division of Technology and Innovation, Intervention Center, Oslo University Hospital, Oslo, Norway

**Keywords:** Research-based teaching, Health professions education, Research process, Learning outcome, Higher education

## Abstract

**Background:**

Integrating teaching and research may boost students’ learning and improve future clinical practice when incorporated into education. Explorations of health professions students’ involvement in the research processes and their learning outcomes are sparse. Thus, the purpose of this scoping review is to explore the existing scientific literature on courses involving students from health professions education in research activities. The research questions are: Which parts of the research process are the health professions students involved in, and what are the students’ main learning outcomes related to the research process reported to be?

**Methods:**

A scoping review following the six-step approach of Arksey and O’Malley was undertaken. We searched four electronic databases to identify studies focusing on research-based teaching in health professions education. Inspired by content analysis, we identified key concepts relating to the research process and learning outcomes.

**Results:**

We screened 1084 abstracts, reviewed 95 full-text reports, and included 24 for analysis. Overall, the students were more involved in conducting and disseminating research than in the planning phases. Learning outcomes were most frequently reported as specific research skills, such as conducting literature reviews, writing academically, and presenting results, but also as improved understanding of research in general as well as improved motivation and confidence in conducting research.

**Conclusions:**

The heterogeneity of educational programs, study designs, and measures makes it difficult to draw conclusions across the studies included in the review. More research is needed to conclude whether health professions students who actively engage in research gain a better understanding of the research process, become more likely to pursue research in their practice, or are more motivated to choose an academic career.

**Supplementary Information:**

The online version contains supplementary material available at 10.1186/s12909-023-04615-4.

## Background

The interplay between research and teaching in higher education is often referred to as a “nexus,” suggesting that the linkage is close, essential, and undeniable [1]. The much-referenced model of Healey [2] differentiates between research-led, research-oriented, research-tutored, and research-based teaching. Research-led and research-oriented teaching are both categorized as teacher-focused, with teaching structured around subject content and methods for knowledge production, respectively. Research-tutored and research-based teaching are presented as student-focused strategies, where the former involves students’ writing and discussions about research, and the latter actively involves the students in doing research [2]. According to Healey [3], a research-based curriculum is preferred because it treats learning as problems that remain to be solved through inquiry and research. Another way of illustrating the variations in linkages between teaching and research is to focus on relationships between the two and on student involvement, presented as a continuum from no relationship between teaching and research and students acting as consumers at one end, and complete integration with students as producers of research at the other [4]. Active student involvement is proposed as fundamental for learning [5]. Student participation in research corresponds with Healey’s description of research-based teaching and is thus recommended for implementation in higher education [6]. Arguments have been made to extend the term “teaching–research nexus” to “the teaching–learning–research nexus” or “the research–education nexus,” including not just the activities of the staff and students but also organizational, institutional, and cultural aspects [6]. In the present scoping review, we maintain the traditional term, as this is commonly used in the literature [7], but with the intention of investigating how health professions students are actively involved in research activities in their educational programs.

The linkage between research and teaching has been shown across disciplines, educational levels, academic orientations of study programs, and characteristics of students [5]. Traditionally, while the teaching–research nexus has been related to study disciplines such as medicine, the concept is increasingly included in programs for applied health studies [1, 8]. There has been a call for more creative and interactive strategies to make research relevant to the practice of nursing [9] as well as occupational therapy and physiotherapy [10, 11]. Many applied programs are at the bachelor level, such as nursing and physiotherapy, and among students in such programs, attitudes toward bringing research into teaching and learning activities have been reported as varied and ambiguous [12]. One issue raised by students is that time spent on research can be at the expense of practical training in profession-specific skills [13]. However, it has been argued that the ability to understand and be involved in research is of great importance to prepare students for a professional career in a rapidly changing, increasingly complex society [14].

Investigations of the relationship between teaching and research are longstanding and have been increasing over the last few decades [15]. However, our preliminary searches revealed a lack of scoping or systematic reviews and a paucity of studies that describe research-based teaching strategies or programs in the breath of health professions educations. Explorations and discussions of the students’ involvement in research processes and their learning outcomes of specific courses were sparse. Thus, we present a scoping review to map and identify available studies and obtain an overview of the topic. The overall purpose of this scoping review is to explore the existing scientific literature on courses involving students from health professions education in research activities.

## Method

This scoping review applies the approaches promoted by Arksey and O’Malley [16], which consist of six stages: (1) identify the research question, (2) identify relevant studies, (3) select the studies, (4) chart the data, (5) summarize and report the results, and (6) consult with stakeholders [16]. The decision was founded on the purpose of examining the extent, range, and nature of the research activity for our topic, to summarize and disseminate research findings, and to identify research gaps in the existing literature [16, 17]. The Preferred Reporting Items for Systematic Reviews and Meta-Analysis for Scoping Reviews (PRISMA-ScR) [18] criteria guided the reporting of the review.

### Identifying the research question

Based on the previous research presented in the introduction and our curiosity as scholars in the field of health education and research, we aimed to answer the following research questions: Which parts of the research process are the health professions students involved in, and what are the students’ main learning outcomes related to the research process reported to be?

### Identifying relevant studies

To identify literature relevant to our research questions, key concepts and terms were developed from the literature relating to the research–learning or teaching nexus. The Norwegian Act for Health Personnel, which corresponds with other European countries on the recognition of professional qualifications, was searched to identify relevant health professions [19]. Health professions were combined with versions of the research–teaching concept. A search string was built and tailored to each database, searching for terms in titles, abstracts, keywords, and MeSH terms. To cover both education and health literature, we searched MEDLINE, Education Resources Information Center (ERIC), and SCOPUS. The first 300 papers listed in a Google Scholar search were also included. Table 1 provides the full search strategy for one of the databases.

**Table 1:** Key search terms (ERIC)

Search terms	
(((DE “Nursing Education”) OR (DE “Medical Education”) OR (DE “Pharmaceutical Education”) OR (DE “Health Education”)) OR health OR med OR nurs* OR physiotherap* OR “physical therapist” OR bioengineer OR ergotherapist OR ergonomist OR occupational therap* OR midwife OR nutritionist OR dietitian OR psychologist OR radiographer OR dentist OR (dental AND surgeon) OR (social AND educator) OR pharmac* OR chiropractor OR optician) AND (“Research teaching nexus” OR “teaching research link” OR “research-based teaching” OR “research-based learning”)	

We included studies reporting on health professions students and research-based teaching as main concepts and excluded studies reporting on evidence-based practice or problem-based learning only. We searched for studies focusing on research activities connected to a specific course or subject excluding studies reporting solely on students’ experiences related to their individual bachelor’s or master’s thesis. All professions not requiring higher education and professions requiring specialization or further education beyond qualification were excluded. A full list of the inclusion and exclusion criteria is outlined in Table 2. Studies after 2000 were searched to include reports published after the initiation of major university reforms in the Nordic countries [20]. The first search was completed in April 2020, with a supplementary search in November 2022.

**Table 2:** Inclusion and exclusion criteria

Criterion	Inclusion	Exclusion	
Study focus	Research-based teaching/learning courses or strategies	Evidence-based practice only Problem-based learning only	
Population	Students or courses (undergraduate/graduate, bachelor/masters level) in health professions education or any course leading to a primary health professions qualification Health professions education, including students in medicine, nursing, physiotherapy, bioengineering, occupational therapy/ergotherapy, midwifery, nutrition, dietetics, psychology, radiography, dentistry, pharmacy, chiropractic, opticianry	Non-students, not health professions education, not higher education	
Outcome	Any kind of student outcome related to research engagement, learning from or through research involvement	No student learning outcomes	
Type of articles	Original, peer-reviewed research		
Language	English or Scandinavian languages		
Time period	January 2000–November 2022		

### Study selection

Identified records were imported into the Covidence systematic review software [21], and duplicates were removed. Random pairs of two independent reviewers screened titles and abstracts for eligibility. Relevant reports were retrieved and assessed in full text against the inclusion criteria. Full-text reports that did not meet the inclusion criteria were excluded, and the reasons for exclusion were registered. Disagreements between the two reviewers were resolved through discussions with a third reviewer. The reference lists of the initially retained reports were hand searched. The selection procedure for the reference reports was the same as described above.

### Charting the data

A data form was developed in Microsoft Excel to extract the data. The following key items of information were obtained from the studies: author, year of publication, location, student sample, aim of the study, methodology, outcome measure, and key results. The included studies were divided equally between the authors, and the data charting was conducted individually before all the authors agreed on the design and content of the final form.

All studies included in the final review were uploaded in full to NVivo [22], facilitating the analysis. Inspired by a directed content analysis approach [23], we identified key concepts related to the research process. We coded text from complete reports pertaining to the main steps of research: *planning the research* (choosing the topic, aims, and/or objective, conducting a literature review, designing the study), *doing the research* (collecting and analyzing the data), and *disseminating the research* (reporting and presenting the results). The coded text was extracted and organized in a table. Furthermore, we extracted all text relating to the students’ main learning outcomes, and using content analysis, we identified the following themes: *knowledge and skills* and *engagement and satisfaction.*

### Consulting with stakeholders

According to Arksey and O’Malley’s sixth stage [16], we presented our review and findings with two stakeholders, both of whom were health care professionals (nurse and physiotherapist), and researchers and teachers with extensive pedagogical and didactical expertise. The stakeholders read through the entire manuscript, provided written feedback on the presentation of the main findings, and suggested relevant issues for discussion. The comments were included in the authors´ deliberations of the presentation of the results and in the discussion of the results.

## Results

### Summary of the studies

**Fig. 1:**
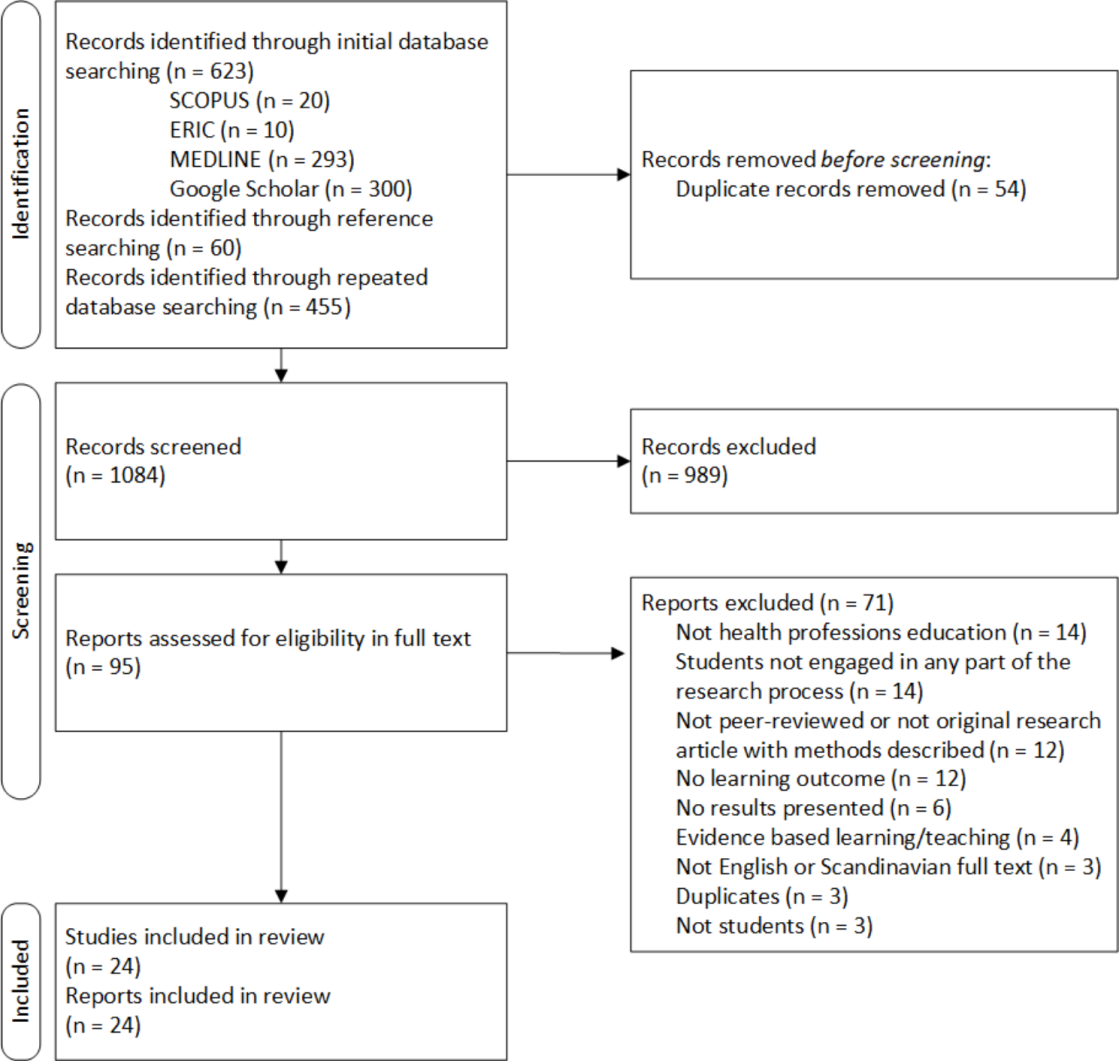
PRISMA flow diagram for study selection

Using the key search descriptors, we identified 1078 records. Through hand searches of the reference lists of the initially retained records, 60 additional records were found and assessed adding up to 1138 identified records in total. Among the records from the search, 54 were duplicates. We screened 1084 records of which 989 were deemed irrelevant. Altogether 95 reports were retrieved and assessed in full text including the 60 records identified through reference searching, and finally 24 reports were included. Figure 1 illustrates the process of article selection.

The eligible studies represented 12 countries (Additional file 1), with the majority (n = 7) being from the United States. The studies covered six different health professions (medicine, nursing, dentistry, pharmacy, psychology, and physiotherapy) in addition interdisciplinary health education programs. Medicine was the most frequently studied health profession (n = 10, 42%). The majority of the studies reported on research-based teaching projects implemented in existing courses, most of which were public health or community health courses. Most studies had a quantitative design (n = 15, 63%), only two (8%) had a qualitative design, and the remaining seven studies (29%) used a multi- or mixed-methods design with both quantitative and qualitative methods. The majority of the studies (n = 21, 86%) included some kind of survey to assess outcomes, of which all but one [24] were designed to fit the specific study context. The surveys asked questions relating to the students’ learning outcomes, perceived involvement, and experience with research participation. There were also examples of studies reporting on achievements (e.g., awards, scholarships, and grants) and scientific production [25–27]. The qualitative studies included interviews, observations, narratives, and text and document analysis [28–31].

### Summary of the students’ involvement in the research process

In all the studies, the students were actively engaged in conducting research, either by participating in data collection or interpreting it, or both (Table 3). In all but one of the studies [29], the students were involved in disseminating the research through the presentation of their results with a written report, poster, or oral presentation. The students were less engaged in *planning the research* than they were in later phases of the research process. The topics for the students’ projects were mostly predetermined; however, there were examples of studies in which the students themselves chose a research topic [26], chose or voted on a topic within an overarching theme [24, 28, 32, 33], or were given the opportunity to choose between several predefined topics [34]. Arguments for letting the students participate in the choice of research topic were to increase enthusiasm and interest [24, 28]. In 11 of the studies, the students were involved in drafting an aim or objective or a research question for their research project, and in 12 studies, students performed a literature review either prior to or after the identification of a research question. In almost half of the studies, the students were involved in the choice of study design.

**Table 3:** Students’ involvement in the different stages of the research process

Author	Year	Choosing a research topic	Drafting aims/objectives	Review of literature	Design	Data collection	Analysis/ interpretation	Report writing	Presentation of results
Balakas [34]	2010	X	X	X		X	X		X
Bertrand [33]	2020	X		X		X	X	X	X
Bouhaimed [35]	2008		X		X	X	X	X	X
Chaturvedi [36]	2001		X	X	X	X	X	X	X
Choeisuwan [37]	2015		X			X	X		X
DeHaven [38]	2005				x	X	X	X	X
DeHaven [39]	2011					X	X	X	X
Deonandan [40]	2013		X	X		X	X		X
Dongre [31]	2011					X	X		X
Eley [25]	2015	X	X	X	X	X	X	X	X
George [41]	2017			X		X	X	X	X
Hardway [24]	2014	X	X		X	X	X	X	X
Hassan [28]	2013	X	X	X	X	X	X		X
Jutlla [29]	2014					X	X		
Kongkaew [42]	2019				X	X	X	X	x
Millar [43]	2009					x	X	X	
Mullan [32]	2014	X	X	X	X	X	X	x	X
Naug [44]	2012				X	x			X
Oakes [30]	2014					X		X	
Smith [26]	2001	X	X	X	X	x	X	X	X
Tamariz [27]	2017		X	X	X		X	X	X
Vereijken [45]	2018		X			X	X	X	X
Veses [46]	2020			X		X	X		X
Wesselborg [47]	2019			X		X	X		

### Summary of the learning outcomes

The majority of the studies measured the students’ self-reported learning outcomes, but there are examples of studies that included assessments of the students’ research skills and knowledge of research methods as well as their academic success (Additional file 1). Students reported increased *knowledge and skills*, such as team skills [33, 40, 44], reading and writing skills, and research and presentation skills [24, 31, 32, 40–42, 45]. Positive outcomes were associated with learning how to make long-term plans and to work systematically [37], to engage in the scientific and creative process of designing, conducting, and implementing research [38], and to conduct literature reviews, write academically, and publish reports [26]. Two studies reported that research-based sessions encouraged critical thinking and reflective practice to support deep learning [29, 46]. Research-based teaching was also reported to increase students’ awareness of the research culture of the faculty and their understanding of academic life [44]. Several of the studies included poster presentations, conference participation, and papers published by students as objective measures of academic output (e.g., 26, 27, 33). One study described how students found it more useful to write and present posters than to write a paper [40]. Factors related to poorer learning outcomes were, for example, unsuitable timing of the course in the program [36] and insufficient preparation for using statistical analysis software [38]. Courses with tight deadlines, that were too time consuming, or had overly complicated instructions were regarded as less useful [26, 37].

Most studies included some kind of measure of students’ *engagement and satisfaction* with research-based teaching. In one study, students reported that it felt purposeful to conduct real research and be able to transfer their findings to practice [34]. Participating in a research project positively affected the students’ confidence in and understanding of research, and the students found it rewarding to be taken seriously as researchers [33, 34, 41]. One study showed that students who knew more about research at the beginning of the course had marginally more positive attitudes initially, but the pre-course differences disappeared by the end of the course [24]. Here, the students’ attitudes toward research were positively related to their overall number of skill-based experiences [24]. Several studies found that research-based teaching increased students’ motivation to participate in research in the future [24, 31, 36, 40]. However, one study showed that even though research might be seen as important for future careers by students, a more research-based curriculum did not affect their beliefs about the value of research [45]. Less engagement in research was grounded in a belief that participation was not contributory for postgraduate courses [36], or was not experienced as sufficiently relevant [30]. In some studies, the students reported that they valued learning about the topic and interacting with the patients in the project more than participating in the research process [30, 47].

## Discussion

The present scoping review aimed to explore scientific literature reporting on specific courses in health professions education in which students were actively engaged in research activities, that is, research-based teaching. We identified and summarized which parts of the research process students were involved in, and what their learning research-related outcomes were reported to be. Overall, the students were notably more involved in conducting and disseminating research and less involved in the planning phases. In some studies, the learning outcomes were reported as improved knowledge and understanding of the research process in general, but most frequently, the studies reported on how participating in research-based courses or programs increased specific research skills. How involvement in research contributed to learning about specific topics was less extensively discussed in the studies and is not within the scope of the present review.

During the screening process, we excluded many studies that reported on courses in evidence-based practice or programs engaging students in learning activities that can be characterized as problem-based learning. Although evidence-based practice and problem-based learning use research evidence and allow extensive student activity, compared to research-based teaching, they do not include activities in which the students take an active part in the research process and learn as researchers [2, 3, 8]. The inclusion/exclusion process confirmed our presupposition that studies on *using* research far outnumbered studies on *doing* research in health professions education. The relevance of evidence-based practice and problem-based learning skills for health professionals is highly acknowledged, and it is established that all health professions graduates should be able to gain, assess, and apply research-based knowledge in practice [48, 49]. Knowing about the research process is important for students in their health professions education and beyond. However, knowledge and experiences acquired through actual training in planning, doing, and disseminating research may add greater value, even if the students´ acquired learning of research is limited to one project.

To a large extent, the research projects included in our review were minor student projects defined and limited by the topic of the course. The results show that students were actively involved in data collection, interpretation of data, and dissemination of research results. It is interesting that some of the courses also managed to involve the students in the initial research phases of deciding on the topic, objective, and design. Providing students with choices and opportunities for self-initiation might support their autonomous motivation and perceived competence [50]. Research has shown that adopting an autonomy-supportive teaching style, for example by issuing a meaningful rationale for the learning activities, and providing choice and involving the students in the course design, may increase their motivation [51]. Several of the included studies reported on motivational outcomes such as satisfaction, engagement, attitudes, or perception of relevance. However, the wide variation in designs across the studies makes it impossible to compare the impact of self-determination on student engagement and learning outcomes. Investigating the motivational effect of autonomy support in research-based courses is an intriguing issue that could be explored in future research.

The included studies typically aimed to measure the impact of a research-based course by comparing perceptions of knowledge and skills or research engagement before and after course participation. With one exception [24], all of the studies designed their own surveys, but included limited information on how the surveys were developed and evaluated. Without proper evaluation of reliability and validity, we cannot ensure that the instruments used were measuring what they were supposed to measure. Moreover, the use of tailored surveys designed to report on the impact of one specific program or course makes it impossible to compare improvements in knowledge of or motivation for research across studies. Thus, in future studies, systematically developed and validated instruments to measure constructs such as students’ attitudes toward research should be applied. The revised Attitudes Towards Research Scale [52], applied in Hardway and Stroud [24] and developed to measure perceptions of usefulness, anxiety toward, and positive feelings regarding research courses, is one, if not the only, instrument designed for this purpose. The scale contains factors measuring attitudes investigated in several of the studies in the present review, such as the value of doing research for its own sake, for practicing for a future research career, or to support practice. The latter conception of research as useful for practice may be of particular importance. Several of the included studies described that students were concerned that research engagement would take time away from learning about a topic and practicing skills, findings that are in line with previous literature [13].

The great majority of courses in the included studies were public health and community health courses. This demonstrates that public and community health are more versatile, relevant, and easy to access for student research than hospitals. In the public health courses described, the students were given the opportunity to engage in research to improve population health outcomes and minimize risks, thereby contributing to reducing health inequities. Rimer [53] argued that to help students focus on achieving a positive impact on health threats, they must be provided with the necessary research skills and tools to identify evidence gaps and be involved in meaningful practice-based research projects. There may also be practical and ethical reasons why research-based teaching is implemented in public health courses and, to a lesser degree, in clinical courses. Particularly in an educational context, investigating population strategies to promote health and prevent disease is less sensitive and ethically demanding than approaching vulnerable patients undergoing treatment.

This scoping review has some limitations. We searched only for studies in peer-reviewed journals and not gray literature. Thus, it is not possible to determine whether our findings are representative of research-based approaches in higher health education. We have reported on studies focusing on research activities connected to a specific course or subject. and did not include studies solely reporting on students’ experiences of doing research related to their bachelor’s or master’s theses. The choice was taken to narrow the scope of our review, but we acknowledge that we may have missed relevant information on how students‘ acquire research experiences from their thesis work. Even though the time span of our scope was more than 20 years and included a wide range of health professions, we found only 24 studies that matched our criteria. The updated search revealed no new articles published during the two years from the first to the updated search. This is likely a consequence of a demanding teaching situation during the Covid-19 pandemic. Research-based courses that require extra resources, as well as access to patients and communities, have been deprioritized [54, 55]. A scoping review does not include a quality assessment of the research included. However, we are left with the impression that the validity of several of the studies was compromised by using unvalidated measures, no control groups, small samples, and limited follow-up times. During the selection process, a large body of research was excluded due to the lack of a clear description of methods or measures to report on the learning outcomes. It is a paradox that articles reporting on research-based higher education courses have extensive methodological shortcomings.

The present scoping review cannot make statements about the overall impact of research-based teaching on students’ knowledge of doing research nor future engagement in research activity. Thus, more research is needed to investigate whether health professions students who actively engage in research have a better understanding of how to conduct evidence-based work, are more motivated to choose an academic career or are more likely to pursue research in their practice. The latter is particularly important as evidence suggests that there is an association between individuals’ and healthcare organizations’ research engagement and improvements in healthcare performance [56]. A recent review found that clinical academic activity may have positive impacts for patients, beneficial impacts to the individual clinical academic, impacts for service provision and workforce, and the organization’s research profile, culture, and capacity, as well as economic impact and impacts on staff recruitment and retention [57].

## Conclusion

In this scoping review, we identified scientific literature on research integration in health professions education. We aimed to investigate students´ participation in different phases of the research process and the learning outcomes reported. We found that in most studies, the students were involved in a range of research activities, but more often in conducting and disseminating the research than planning it. Reported learning outcomes included improved research skills, such as conducting literature reviews, writing academically, and presenting results, as well as increased motivation, confidence, and understanding of research. However, the heterogeneity of educational programs, study designs, and measures makes it difficult to summarize the outcomes. Understanding how students can be involved in research and exploring learning outcomes related to such research-based strategies appears to be crucial in enabling the development of educational programs for health professions students.

### Electronic supplementary material

Below is the link to the electronic supplementary material.

**Additional file 1:** Summary of included studies

## Data Availability

Not applicable. All data were drawn from published manuscripts.
