# Supplementary material for: Integrating research in health professions education: a scoping review
Source: BMC Med Educ. 2023 Sep 8;23:653. doi: 10.1186/s12909-023-04615-4 (PMC10492286; doi:10.1186/s12909-023-04615-4)
Supplement: Supplementary file 1 — Additional file 1: Summary of included studies [file 12909_2023_4615_MOESM1_ESM.docx]

**Additional file 1.** Summary of included studies

| Author | Year | Location | Student sample | Aim | Methodology | Outcome measure | Key results |
| --- | --- | --- | --- | --- | --- | --- | --- |
| Balakas | 2010 | USA | Nursing  N = 75 students, 5 students each group, 15 groups  Health promotion | To describe and evaluate a project giving undergraduate nursing students the experience of participating in research in collaboration with the practice field. | Mixed method | Students’ perceptions of value and outcome of participation (questionnaire, including open-ended items) | Positive experience, achieving learning outcomes of the course, increased interest/motivation for evidence-based practice and research was found. |
| Bertrand | 2020 | USA | Interdisciplinary  N = 51,  11 teams  Community health | To develop an expanded SHP honors curriculum that would provide undergraduate students with an integrative, real-world problem-solving experience in the broad arena of healthcare and healthcare disparities within the local community. | Mixed method | Students’ perceptions of their learning (questionnaire) | High student satisfaction with achieving learning outcomes was reported. |
| Bouhaimed | 2008 | Kuwait | Medical  N = 104 project groups  (Over 5 years)  Public health | To evaluate the factors related to quality, impact, and relevance of a 6-week student project for teaching public health. | Quantitative | Quality, impact, and relevance of student projects (questionnaire/checklist) | Various factors associated with improved quality, impact, or relevance to public health practice, e.g., increasing departmental experience in handling student projects and nation-wide research. |
| Chaturvedi | 2001 | India | Medical  N = 314,  11 groups  Community health | To document learners' feedback on an educational intervention to provide interns with hands-on learning experience in population-based research. | Quantitative | Students’ participatory involvement (questionnaire) | Participatory involvement rated as satisfactory, good, or maximal on most learning units by the majority. Report writing and presentation got the lowest scores, data collection, identification of research question, review of literature, and data analysis got the highest. |
| Choeisuwan | 2015 | Thailand | Nursing  N = 80  Nutrition and health | To study the ability of the nursing student in conducting research, to compare learning achievement before and after the research-based instruction in the topic of nutrition and health and to investigate the nursing students' opinions toward the instruction. | Quantitative | Learning achievements in nutrition and health, ability to conduct research, opinions about instruction (questionnaires) | Research-based instruction enhanced the students’ abilities in teamwork, systematic work, analytical thinking, and synthesizing. |
| DeHaven | 2005 | USA | Medical  N = 13  Community health | To develop and evaluate the effectiveness of a training program for teaching medical students research, while performing community-based participatory research in an underserved area. | Quantitative | Program evaluation (questionnaire, added open-ended items) | The students reported improved understanding of the research process. |
| DeHaven | 2011 | USA | Medical  N = 2–7 students annually, in total  25 completed projects  Community health | To provide an overview of the Community Health Fellowship Program, describe the types of projects, and address the program’s effectiveness. | Quantitative | Evaluation of the curriculum (questionnaire) | The students reported improved understanding of research process and attentiveness of underserved communities. |
| Deonandan | 2013 | Canada | Interdisciplinary  N = 80  Epidemiology | To implement teaching that exposes students to realities of scientific life, improve their abilities to make decisions on their career path, and apply and enhance presentation and communication skills. | Quantitative | Students’ perceptions of knowledge improvements and usefulness of the assignment (questionnaire) | Almost 20% and 30% reported that they were more likely to pursue a career in science and epidemiology, respectively. A majority reported that oral poster presentations were useful. |
| Dongre | 2011 | India | Medical  N = 55  Public health | To investigate the effect of exposure to field surveys in the training of community medicine on medical undergraduate students. | Mixed method | Student skills (questionnaire, retrospective post-then-pre and open-ended questions after 1 year) | The students reported that they became sensitized to the importance of the use of structured data collection and analysis. |
| Eley | 2015 | Australia | Medical  N = 42  Multiple research aeras | To address the decline in clinician scientists by increasing the number of students on a formal pathway to an academic research career, and building a “teaching–research nexus” using the research-intensive environment at the university. | Quantitative | Publications, research presentations, awards, grants (number). Future research plans and interests (questionnaire) | Increased student progression, outstanding students won scholarships (76%), increased number of publications and conference presentations was reported. |
| George | 2017 | USA | Physical Therapy  N = 10  Community health | To describe the impact of participation in 2 interdisciplinary community-based research projects. | Mixed method | Learning outcome (questionnaire) | The students reported that research skills and academic writing skills increased because of participation. |
| Hardway | 2014 | USA | Psychology  N = 47  Student life  Topic of interest | To examine whether the curriculum was associated with an increase in students' knowledge of research methodology, their attitudes toward the research process and their professionally related experiences. | Quantitative | Students’ knowledge of research methods, attitudes toward research and skill-based experiences | Significant gain in skill acquisition, knowledge of research methods, and improved attitudes toward research was found. |
| Hassan | 2013 | UK | Medical  N = 80  Special study modules | To describe and discuss the implementation of research-based learning through special study modules in the undergraduate medical program at a university in the UK. | Qualitative | Observation of lectures and workshops; document analysis of handbook, journals, posters, and correspondence; time used | The students acquired knowledge and skills related to research processes and produced knowledge on a chosen topic. |
| Jutlla | 2014 | UK | Health and social care  N = 16  Dementia care | To explore the teaching–research nexus by using a research-based approach. | Qualitative | Qualitative analysis of storyboards and group discussions | Deeper student learning supported by increased intrinsic motivation, critical thinking, and reflective practice were found. |
| Kongkaew | 2019 | Thailand | Pharmacy  N = 83  Pharmacoepidemiology | To test the impact of research-based learning on student knowledge and assessment in pharmacoepidemiology to prepare pharmacy students for their future roles in society and ultimately contribute to health care and patient safety. | Quantitative | Pre- and post-tests on students´ knowledge of observational and experimental study design, and questionnaire assessing teaching and student activity | The students reported increased knowledge on observational and experimental study design and high ratings on teaching quality. |
| Millar | 2009 | New Zealand | Medical  N = 136  Public health | To consider the feasibility and perceived value of an integrated teaching/research approach. | Mixed method | Students´ course evaluation (questionnaire) | Most students responded favorably to the teaching exercise with raised awareness and interest in public health and research. |
| Mullan | 2014 | Australia | Medical  N = 165  Public health | To examine whether the self-perceived research experiences of medical students, and consequent research capability, were influenced by exposure to an innovative research and critical analysis curriculum. | Quantitative | Self-assessed levels of research experience (questionnaire) | Improved self-perceived research experiences among students and positive impact on students’ research capabilities was reported. |
| Naug | 2012 | Australia | Interdisciplinary (health disciplines)  N = 641  155 groups of 4–5 students  Faculty-based research aeras | To demystify health-related research and provide students with first-hand experiences of the research culture. | Quantitative | Students' interest for and awareness of faculty research and higher education programs (questionnaire) | The students reported positive experiences with research encounters and enhanced engagement in investigation process. Research career aspirations were unchanged. |
| Oaks | 2014 | USA | Health profession education  N = 18  Community health | To describe a service-learning project that was designed to help undergraduate health professions students the complexities related to aging in place. The service-learning project also incorporated a research component to expose the students to  the research process. | Quantitative/ qualitative | Perception of learning experiences (questionnaire and narratives) | The students valued one-on-one interaction with older adults more than participating in the research process. |
| Smith | 2001 | Canada | Medical  N = 63  Faculty-based research aeras | To outline the development and evaluation of the Research Project Program (RPP) 10 years after its introduction into the medical curriculum at the University of Calgary. | Quantitative | Experiences and research activities (questionnaire) | Compared to year one, most of the students from year 10 were engaged in research projects, the majority had presented their research, and more than half were planning to submit research, but the program did not get high ratings from the students. |
| Tamariz | 2017 | Ecuador | Medical  N = 50  15 groups  6–7 members per team, including trainees/professors  Multiple topics | To report the experience of adapting an active learning-based research methods curriculum to improve research productivity at a university in Ecuador. | Mixed method | Scholarly success (presentation of group research results), readiness for research (questionnaire), satisfaction with the course (interviews) | The students reported increased knowledge and perceived competence in the research process, high course satisfaction. Presentation rate was 50% for the group. Barriers to research were curriculum and lack of research culture. |
| Vereijken | 2018 | Netherlands | Medical  N = 746  Epidemiology | To determine whether a curriculum change intended to promote research integration fosters student learning outcomes and student perceptions of research integrated into teaching. | Quantitative | Cognitive learning goals (national progress test), student performance (rated research abstract), students’ perception of research integration (questionnaire) | Increased scores on research-related test items and increased quality of test research reports were reported. The curriculum improved the perception of research integration but did not affect beliefs about the value of research. |
| Veses | 2020 | Spain | Dental  N = 10  Microbiology and virology | To evaluate the implementation of a student-led, research-based strategy to teach non-laboratory elements of the microbiology curriculum for dentistry, specifically the increase in anti-microbial resistance. | Quantitative | Achievement of learning objectives with 2 questions relating to the research process (questionnaire) | Improvement in dental knowledge and ethics in biomedical research (student ambassadors and students), improved knowledge about critical thinking and study design (student ambassadors) was reported. |
| Wesselborg | 2019 | Germany | Nursing, medical  N = 46  Nutritional  management | To test an interprofessional training session on the topic of malnutrition using the method of research-based learning to evaluate the feasibility. | Quantitative | Application of research-based learning, social climate, relevance and course satisfaction (questionnaire) | The social context and relevance of the topic were rated very highly, use of research-based learning was rated as satisfactory. |
|  |  |  |  |  |  |  |  |
